# What do stroke survivors want in stroke education and information provision in Australia?

**DOI:** 10.1111/hsc.13896

**Published:** 2022-06-29

**Authors:** Emma Finch, Ellie Minchell, Ashley Cameron, Katherine Jaques, Jennifer Lethlean, Darshan Shah, Christian Moro

**Affiliations:** ^1^ School of Health and Rehabilitation Sciences The University of Queensland Brisbane Queensland Australia; ^2^ Speech Pathology Department Princess Alexandra Hospital, Metro South Health Woolloongabba Queensland Australia; ^3^ Centre for Functioning and Health Research, Metro South Health Woolloongabba Queensland Australia; ^4^ Clinical Support Services, Metro South Health Woolloongabba Queensland Australia; ^5^ Division of Medicine Princess Alexandra Hospital, Metro South Health Woolloongabba Queensland Australia; ^6^ Faculty of Health Sciences and Medicine Bond University Robina Queensland Australia

**Keywords:** consumer feedback, end user, focus group, patient perspective, qualitative, stroke, stroke education

## Abstract

Despite evidence that stroke education is important and effective, low rates of stroke education are reported worldwide. Many stroke survivors and carers report that current stroke information does not meet their needs. The aim of the current study was to explore the perceptions of stroke survivors and carers towards stroke education in an Australian health context. A qualitative descriptive approach using focus groups was used to explore education received and preferred content, format and timing of education. Data were analysed inductively using qualitative content analysis to identify key categories, sub‐categories and an overarching theme. Fifteen stroke survivors and four carers participated. Four categories emerged: the timing of stroke education, the content of stroke education, the format of stroke education, and reactions to stroke education. Each category contained a number of sub‐categories. One overarching theme was woven through the transcripts: everyone is different and has different needs. Overall, participants generally expressed positive attitudes towards stroke education. Participants reported that education should occur at multiple timepoints and in a mixed format. All participants reported receiving stroke education in hospital, but not in the community. Stroke survivors preferred group education, while carers did not share this preference. Both stroke survivors and carers desired information about post‐stroke physical changes and computer use; however, stroke survivors desired additional information spanning a variety of areas such as motivation and driving. Tailoring of stroke education for each individual is critical to ensure that education meets the needs of stroke survivors and carers from the hospital to the community.

**What is known about this topic?**

- Stroke education is valued by stroke survivors and carers.
- Rates of stroke education remain low globally.

**What this paper adds?**

- There is no one‐size‐fits‐all for stroke education.
- Information about secondary stroke prevention can be perceived negatively by stroke survivors, and therefore should be tailored to the individual.
- While stroke survivors and carers desire stroke education in mixed formats at multiple timepoints, stroke survivors find group formats beneficial.
- Stroke survivors desire stroke education content in a large number of areas beyond those desired by carers.

## 1 INTRODUCTION

Stroke is a leading cause of death and disability globally (Johnson et al., 2016). In the United States, more than 795,000 people experience a stroke annually (Centres for Disease Control and Prevention, 2017), while in the United Kingdom over 100,000 people experience a stroke (Stroke Association, 2017). In Australia, it is projected that the number of strokes per year will increase to over 130,000 by 2050 (Stroke Foundation, 2017b). Once an individual has a stroke, they have an accumulated risk of subsequent stroke of 43% over 10 years (Hardie et al., 2004). This high risk of subsequent stroke places a substantial burden on community‐based healthcare. Stroke education, that is information about the mechanisms of stroke and stroke prevention, is, therefore, a vital component of stroke management. In recognition of this, stroke education features in stroke clinical guidelines internationally (e.g. AUS—Stroke Foundation, 2018, USA—Powers et al., 2019, UK—Royal College of Physicians, 2016).

It is important to note the delineation between stroke education and stroke information, with stroke education referring to a structured active approach to information provision and stroke information referring to the more passive giving of information. A body of research has explored the benefits of stroke education and stroke information. A Cochrane review by Forster et al. (2012) found that stroke information improved stroke survivor and carer knowledge, elements of stroke survivor satisfaction, and reduced stroke survivor depression. Activity limitations, participation and service use did not significantly change (Forster et al., 2012). The review focused on information provision, with active provision (i.e. education) noted to promote better outcomes than passive information provision. A subsequent Australian study by Eames et al. (2013) found that stroke education using the strategies of a computer‐generated individually tailored written information book and verbal reinforcement (provided over 3 months) significantly improved self‐efficacy for accessing information and satisfaction with information received compared to standard care (which involved informal verbal education provided ad hoc as part of routine care by health professionals). More recently in Taiwan, Kang et al. (2019) compared a Stroke Health Education Mobile App (SHEMA) with usual care (stroke health education booklet). SHEMA enabled participants to access the same stroke information provided in the health education booklet, but in digital form. Both approaches significantly improved participants' knowledge of stroke risk factors, with no significant difference between the approaches (Kang et al., 2019). It is possible that the difference in outcomes between Kang et al. (2019) and Eames et al. (2013) may have partially reflected the different study foci (i.e. Eames et al., 2013 focused on using two strategies to support education while Kang et al., 2019 focused on comparing app vs. booklet outcomes).

Despite growing evidence that stroke education is beneficial, low rates of stroke education are reported worldwide (Hoffmann & Cochrane, 2009; Stroke Foundation, 2017a); over 75% of stroke survivors and carers report that their information needs are not met (Eames et al., 2003). Despite earlier research into stroke education in Australia (Eames et al., 2011, 2013), a 2021 Stroke Foundation audit reported that almost 1/4 of stroke survivors do not receive stroke risk factor education (Stroke Foundation, 2021a). This is despite initiatives such as the Stroke Foundation's My Stroke Journey, a stroke information booklet launched in 2013 designed to be provided to all stroke survivors in Australian hospitals (Stroke Foundation, 2019, 2021b). This suggests that there is still a critical need to determine how to maximise stroke education in Australia. Additionally, given the increasing use of technology and changes in acute stroke care, such as reperfusion therapies and hyper‐acute stroke units, there is a need to explore stroke survivors and carer perspectives about stroke education in a contemporary healthcare setting.

The aim of our project was to explore the perceptions of stroke survivors and carers towards contemporary stroke education. With the growing value of consumer feedback into healthcare being recognised (Australian Commission on Safety and Quality in Health Care, 2019; Banfield et al., 2017), seeking the views of stroke survivors and carers is important in bridging the evidence‐practice divide. Increasing our understanding in this area will ensure that stroke education meets the needs of stroke survivors and their carers both nationally and internationally.

## 2 METHODS

### 2.1 Design

A qualitative descriptive approach (as described by Sandelowski, 2000) using a focus group design (as per Rosenthal, 2016) was used to explore stroke survivor and carer perspectives of stroke education.

### 2.2 Participants

Fifteen stroke survivors (seven females and eight males) and four carers (two females and two males) participated. More stroke survivors than carers were included as stroke education is more frequently provided to stroke survivors than carers at the recruiting hospital. Stroke survivors were individuals who had experienced a stroke and were either presently or previously an inpatient at the recruiting hospital (acute and/or subacute). Participants who had been a previous inpatient were still receiving outpatient services through the hospital. Carers were family members or friends currently caring for the stroke survivors participating. Stroke survivors were identified by one of their treating health professionals at the hospital, and carers were identified by the stroke survivor and subsequently recruited. Inclusion criteria were: aged over 18 years and adequate English skills to participate. Participants with post‐stroke communication and cognitive impairments were included if they could communicate with communication support. Ten stroke survivors had been diagnosed with aphasia according to a Speech‐Language Pathologist (SLP) prior to the study. Three of these participants had also been diagnosed with apraxia of speech. None of these individuals were receiving SLP intervention at the time of the study. Time post‐stroke ranged from under 1 month to 4 years. No participants withdrew following study commencement.

### 2.3 Context

Participants were recruited from a single, metropolitan, tertiary hospital in Queensland, Australia. The hospital is a keystroke centre and is part of a health service that provides care for approximately 1.6 million people. The hospital has a hyperacute stroke unit, inpatient rehabilitation unit and outpatient stroke rehabilitation services. Team‐based multidisciplinary stroke management (including medical, nursing, physiotherapy, occupational therapy and speech pathology) is provided. A subset of patients receive inpatient rehabilitation, and or outpatient rehabilitation. If required, stroke survivors may be referred to community or private services. The average acute hospital length of stay for stroke survivors in Queensland is 4 days.

### 2.4 Procedure

Ethical approval was obtained from the relevant Human Research Ethics Committee. Potential participants were identified and informed about the study by their treating stroke professional. All participants provided written consent via either a regular or an aphasia‐friendly Participant Information and Consent Form (PICF, based on the potential participant's preference). The aphasia‐friendly PICF was developed by an SLP within the research team and was designed according to aphasia‐friendly principles previously described in the literature (Rose et al., 2011). Participants attended a single face‐to‐face focus group at the recruiting hospital between November 2018 and June 2019. Two focus groups were conducted, with 6 participants in one focus group and 13 participants in the other. The focus groups were a mix of stroke survivors and carers to maximise discussion of the ideas raised. Each focus group was run by two researchers (EF, EM, JL, AC: all qualified SLPs with clinical and research experience interacting with stroke survivors and as such we are able to provide appropriate communication support for participants with communication difficulties; three of the researchers held PhDs and one was completing a PhD at the time) and lasted for up to 1 h. Only participants and researchers were present during each focus group. Some participants and researchers knew each other before the study, while others had no previous relationship. During each focus group, participants were asked their thoughts about stroke education (see Table 1 for the focus group questions). Focus groups were video recorded for transcription (EM) and analysis. Recruitment occurred until theoretical saturation of the data was reached (i.e. no new information was gleaned).

**TABLE 1:** Focus group questions

Focus group questions
Do you remember receiving any education/information about stroke while you/your family member was in hospital? If yes, can you describe what information was provided and how it was provided? Did you like the information that was provided? Why/why not? Do you think other information would have been useful? What information do you think is very important for people who have experienced a stroke and their families? How do you think the information should be provided? When you do think information about stroke should be provided? Other thoughts?

### 2.5 Data analysis

Transcripts were analysed inductively using qualitative content analysis to identify key categories, sub‐categories and overarching theme(s) in the data (Graneheim & Lundman, 2004). Content analysis was selected instead of thematic analysis to provide information about the frequency of the data to assist with interpretation. During the analysis process, two members of the research team (EF and EM) independently read the focus group transcripts and identified meaning units (i.e. words and sentences that related to the same meaning), then labelled each meaning unit with a code. The two researchers then met to develop a single set of consensus codes. Differences in coding were resolved through discussion. One researcher (EF) then classified the agreed codes into categories and sub‐categories. Data were managed using Microsoft Word. To ensure the credibility of the analysis (as per Graneheim & Lundman, 2004), a number of actions were undertaken. This included sampling participants from multiple groups within the typical caseload at the facility (including recruitment of people with stroke and carers, inpatients and outpatients, people with aphasia and people without aphasia), discussing codes between members of the research team, and use of participants' direct quotes in this manuscript. To enhance transferability, the context (recruiting hospital) is described in detail in the Methods section. Rigour was ensured by the independent coding by two members of the research team and the use of an audit trail and field notes.

## 3 FINDINGS

All participants reported receiving prior stroke education, albeit in varying formats. One overarching theme emerged: *everyone is different and has different needs*. This theme highlighted the importance of varying stroke education to accommodate everyone's individual stroke, demographic characteristics, experiences and needs. This emphasises that tailoring of stroke education for each individual is critical.

Four categories emerged from the transcripts: the timing of stroke education, the content of stroke education, the format of stroke education, and reactions to stroke education (see Table 2). Each category will be discussed below. Note: Participants quotes are labelled FXPY (with X denoting the focus group number and the Y donating the participant number. Carers are identified by underlining).

**TABLE 2:** Categories and sub‐categories from the content analysis

Category	Sub‐category
1. The timing of stroke education	Education started early Education occurred multiple times
2. The content of stroke education	Content received Content desired
3. The format of education	Group and/or individual sessions Mixed format of delivery Use of technology Self‐directed research A reliance on other people
4. Reactions to the stroke education	Positive reactions to stroke education Negative reactions to stroke education

### 3.1 Category 1: The timing of stroke education

The first category, the timing of the stroke education, was an important aspect for participants. This category contained two sub‐categories. The first was that Education started early. Typically participants remembered that education started early, with one participant reporting its occurrence in the *…first few days …* (F1P1). Carers also recalled their significant others receiving stroke education early *When she was in the ward someone, I can't remember who, but someone had to come down and talk to her* (F1P9). Within this category, there was a strong message expressed by all participants that education occurred too early. This included being too early to remember the information *…I don't remember it* (F1P4), not being able to understand the information *…I didn't understand anything for the first I dunno it might have been four days you know it was longer than four days. It might have been 4 weeks. Then from 4 weeks on it slowly started to come back …* (F1P5), not being in an adequate emotional state to receive information *I was not in a fit state to fully digest everything. And uh, it was a bit scary I suppose* (F2P1), and adjusting to the reality of what had happened *Well I was just coming to terms with being asleep for so long that uh it took a while for me to um comprehend that I had uh actually I had ah um bleed in my brain* (F2P2). F1P5 expressed the idea that education may also be too early for family members *Oh my family do without that* (F1P5).

The second sub‐category was Education occurred multiple times. The majority of participants reported receiving stroke education at multiple time points from different health professionals. Five participants (F1P9, F1P6, F1P1, F1P2, F1P5) expressed that stroke survivors and their families should receive information about stroke at multiple timepoints. No participant expressed that stroke education should occur only once. One family member highlighted the value of this:

The biggest first lot of information I got was from the discharge system when she was discharging and it was verbal. But then again half of what I heard I have forgot it. The back up come from there when we started getting literature from the stroke foundation and that's where it all started to come together (F1P9).

Key to the notion of receiving education at multiple time points was that needs are different at different times. For two participants (F2P1 and F2P5), this was verbal information and reassurance from health professionals initially followed by written information. Other participants thought that written information was useful as *…they [can] read which bit they need at that particular time* (F1P8). This echoed the overarching theme of everyone is different and has different needs, illustrating that there is not a one size fits all approach to stroke education.

Interestingly, F1P6 who had experienced two strokes with two different hospital admissions reported that the education differed between the admissions, with less education provided following the second stroke. *…two strokes … the second one … just a bit less [education]*.

### 3.2 Category 2: The content of stroke education

The second category, the content of stroke education, addressed both education sessions content that participants had received and education session content that participants desired. All participants reported receiving stroke education while in hospital (or in the case of the carers while their significant other was in hospital). Information received ranged from general stroke information (F2P5) to personalised information about aphasia (F1P1). Participants were divided about information about second stroke prevention. One participant who had experienced two strokes indicated that after the first stroke minimal information was discussed about the potential of experiencing a second stroke:

Ah no I um this one's my second and um … I had one back in trying to think. Um, October. And I didn't get a lot of information then about you know, what to do or how to prevent it. Um. It ah. So you know? I followed the diet and did a few of the exercises but it wasn't, it wasn't really discussed you know anywhere about you know it can happen again. (F2P2)

In contrast, another participant reported that information received about secondary stroke prevention was *Not good. Very depressing it was. Very depressing* (F2P5). This highlighted the importance of emotional sensitivity when providing stroke education to stroke survivors, particularly given that survivors may be at an emotionally vulnerable time in their lives.

The information participants desired is presented in Table 3. This information covered a broad range of areas covering post‐stroke physical changes, hospital processes, stroke prevention, returning to work, computers, specific‐stroke information and emotional changes. Along with stroke survivors, carers desired information about physical changes after stroke and computer use; however, stroke survivors desired further information in a diverse variety of areas beyond these two areas.

**TABLE 3:** Stroke education content desired by participants according to participant number

Content area	Participant number
The physical changes, for example right‐sided weakness, swallowing	F1P1, F1P11, F1P6, F1P8, F1P7, F1P9
Driving after stroke	F1P3, F1P1
Stroke prevention	F1P3, F2P5
Returning (or not returning) to work and study	P2P5, F1P1
How to use computers	F1P6, F1P5, F1P1, F1P2
Accessible facilities for people with disability after stroke	F1P1
Places that can help me and where to find more information	F2P5
How long it will take to recover and what we can do to speed up the recovery process	F2P5
Going home	F2P1
Motivation after stroke	F2P5, F2P1
The emotional impact of stroke, for example frustration	F2P1
Information about hospital processes	F2P5
Personally specific information about my stroke, for example the percentage of people who have my type of stroke	F2P1, F2P5, F1P1

*Note*: Underlining denotes that the participant was a carer.

### 3.3 Category 3: The format of stroke education

The third category explored participant's perceptions towards the format of stroke education. Participant responses within this category fell into a number of sub‐categories (see Table 2). The first sub‐category was group and/or individual sessions. Group sessions were highly valued by stroke survivors, but not carers. For some people this was due to being able to relate to other people in a similar situation:

It helps to um relate to all of them who have the same problem as I have. That's the main thing and that it makes a lot of difference when you come here and see that there are other people also suffering from the same thing. (F1P1)

Participants valued the opportunity to share feelings during groups:

*…just talking about how we're feeling and how it's affecting us and those day to day things. I found that really helpful …* (F2P5). This participant (F2P5) expressed that the interactive nature of group education sessions was beneficial. While group sessions were highly valued, F2P4 and F2P1 found individual education useful: *They come around and they talk to you and I find that really really helpful. It makes you feel that somebody's there working with you. You're not alone. That is really helpful* (F2P4). This quote highlighted the importance of rapport and individualised education.

While one participant (F1P2) preferred receiving information verbally, the majority of participants (including stroke survivors and carers) favoured a mixed format of education delivery. One participant commented *You've got people of all ages affected differently and everybody has a personal preference. Sometimes you can take in written information and sometimes you can't …* (F2P5). Similarly, F2P1 commented *Well I suppose the problem is that we are all suffering from different types of strokes and problems. Uh, so, um … I mean I could probably sit through a visual presentation but I know that my what's happening to me, might be different to everybody else …*. Six participants (F1P9, F1P5, F1P2, F1P8, F2P1 and F2P5) valued written information as a component of stroke education. Often this was due to difficulty retaining verbal information *I'd like to read about it. It's no good somebody talking to you about it … But if somebody talks to me about it, I won't remember it* (F1P5) and the capacity to read the written information at a later date *It was more sort of later on that I read it* (F2P5). F1P1 expressed that including pictures and music would be helpful. This highlighted that there is no one education approach ideally suited to all stroke survivors and carers.

In terms of use of technology, six participants (F1P6, F1P5, F1P1, F1P8, F1P10 and F1P11) were interested in receiving stroke education via computers. F1P1 thought that computers *…would be much simpler …*, while F1P10 believed that apps would offer a more interactive learning opportunity. F1P2 was concerned that their lack of skills with technology would prevent use of computers for stroke education *…I don't know what to do …* In contrast, other participants (F1P5, F1P1 and F1P11) who had used computers frequently pre‐stroke thought that receiving stroke education via technology would provide an opportunity to re‐learn lost computer skills.

Three participants spoke of using self‐directed research to find out stroke information: *…I'm learning every day. I read everything I can find just to you know—there might be just some other little thing that you know I can do that ah I'm not doing now …* (F2P2). Sources accessed were the Stroke Foundation's EnableMe website (F1P5) and Google searches (F2P5 and F2P2). This highlighted that some participants were using technology to seek out additional information about stroke beyond information that was provided by their health professionals. In addition to these three participants, while all participants reported receiving stroke education from the hospital, only one participant reported receiving information from an additional source (university). No participants reported receiving education in the community, despite several participants being multiple years post‐stroke.

Five participants (F1P7, F1P4, F1P5, F2P1 and F2P3) spoke about a reliance on other people when receiving stroke education. This was usually family members or doctors. For F2P1, he perceived that his medically‐experienced family members were more able to ask appropriate questions and understand answers from the medical team: *I did say to doctor I come from a medical family and I felt awkward about asking medical questions so I I got my doctor son in law to write down his questions*. F1P7 found that her son was more readily able to access stroke information from his phone, which alleviated her concerns: *Because my son phone information about stroke so should he he try to help no I'm not worried too much*.

### 3.4 Category 4: Reactions to the stroke education

The final category addressed participants' reactions to stroke education. Within this category there was a dichotomy of responses between positive and negative reactions. Positive reactions typically related to information being perceived as “good” (F1P4) or being able to relate to the information:

I‐I‐I still remember that photograph over here in the uh foil which they show that one side of uh my uh lip was uh drooling and uh uh I could relate to that. (F1P1)

Two participants (F1P7 and F1P9) spoke of the value of stroke education, particularly with respect to family members being able to use information learnt during previous stroke education when the participant experienced a second stroke:

I think why we come in here in group because of my stroke similar to his and … I got second stroke uh second time my husband knew that so took straight away in so … so very quick … . (F1P7)

Negative reactions came from a sense of shock, devastation, and feeling overwhelmed. For some participants the sense of shock and devastation stemmed from information related to secondary stroke prevention and the involvement of people who had experienced a second stroke as co‐presenters in education sessions:

I mean he was a nice guy and everything else and it's good of him to give his time but you know, it's all about the second stroke and he'd had a second stroke and came out feeling absolutely devastated. We are still trying to come to terms with the fact that we've had a first stroke. So I just found that was a real shock. (F2P5)

Another participant spoke about feeling despair at not understanding information provided *…I feel a bit of despair that the messages aren't getting through …* (F2P4). While another participant commented that the stroke education did not seem important: *you get a fair bit of coverage or at least I did, but it wasn't important to me. It didn't feel important* (F1P5). Interestingly, while both stroke survivors and carers reported positive reactions to stroke education and highly valued the education, only stroke survivors reported experiencing negative reactions.

## 4 DISCUSSION

There is a growing body of evidence supporting the need for stroke information and education for stroke survivors prior to hospital discharge (Eames et al., 2013; Forster et al., 2012; Kang et al., 2019). Despite this, the rates of stroke education are less than ideal (Hoffmann & Cochrane, 2009; Stroke Foundation, 2017a). To help understand this discrepancy, the current study explored stroke survivor and carer perspectives of stroke education. Overall, participants generally expressed positive views towards stroke education but reported that current education does not meet all their needs.

Woven through the transcripts was the theme *everyone is different and has different needs*. This highlighted the importance of accommodating for everyone's individual stroke, demographic characteristics, experiences and needs. The concept of sensitivity also traversed a number of categories. Tailoring of stroke education for each individual is critical, and echoed the findings of the Cochrane review by Forster et al. (2012). In the present study, differences were also evident between stroke survivors and carers in desired content and format of stroke education. Another contrast between participants was that stroke survivors only reported negative reactions to stroke education.

The timing of stroke education was identified as a key category in the present study. Participants recalled receiving information ‘early’ following their stroke; however, multiple participants also reported difficulties with memory and information recall at this time. Family members also commented about struggling to recall information in the early stages. Hogan et al. (2016) suggest that stroke has a significant impact on prospective memory and that the level of prospective memory impairment can be greatly impacted by time since stroke onset. In line with Forster et al. (2012), it suggests that it is critical that stroke education is provided at multiple time points. This idea was valued by participants and is pertinent given that stroke education needs may change over time (Eames et al., 2011). Cumulatively, this evidence suggests that stroke education should not stop when a stroke survivor leaves hospital but should instead continue into the community.

The content of the information stroke survivors desire was also discussed in the focus groups. This detailed content desired by participants was a novel finding, as previous research has restricted content to four content areas (Eames et al., 2011), rather than open‐ended content suggestions. Participants identified the need for careful wording and phrasing of information in this potentially vulnerable time, with some information about secondary stroke prevention described as “very depressing” (F2P5) as they were still coming to terms with having a stroke. This highlights the potential need for redistribution of this education in the early stages post‐stroke. Evidently, it is essential to balance the importance of education about secondary stroke prevention with a sensitive approach to ensure the continuity of hope for stroke survivors.

In the present study, preferences for the format of stroke education were varied. Participants mostly preferred a *mixed format of education* delivery, consistent with previous research (Eames et al., 2011). The provision of written information combined with verbal information could provide stroke survivors with tangible information to re‐refer to in light of potential prospective memory difficulties. In the present study, participants valued receiving education in a group style, as this allowed them to relate to other individuals. As an active provision of stroke education is more effective than passive information provision (Forster et al., 2012), it is possible that group interactions may facilitate more active involvement in stroke education. The present study also revealed interest in using technology for stroke education, with some participants expressing positive support towards using computers. Modern modes of content delivery, such as tablet‐based learning and augmented reality, are increasingly utilised to teach human biology and health (Moro et al., 2020). These interventions may present novel options for content delivery surrounding stroke; however, consideration must be given to patients who are uncomfortable using computers.

Stroke education is more commonly provided to stroke survivors than carers (Prick et al., 2021). However, stroke affects the whole family, not just the stroke survivor. This is important as several stroke survivors in the present study commented on poor memory early after stroke and not being able to take in the information, which is consistent with literature reporting that approximately half of stroke survivors remember information provided while in the emergency department (Prick et al., 2021). Five participants spoke about a reliance on other people (including family members or doctors) when receiving stroke education, highlighting the importance of stroke education delivery to the whole family, rather than only stroke survivor.

Given the potential physical and psychological impact of stroke (Harrison et al., 2017; Sennfält et al., 2019), and increased the likelihood of further strokes (Hardie et al., 2004), it is vital that stroke survivors receive stroke education. This project has identified that stroke survivors value stroke education and has highlighted a need to investigate potential alterations in stroke education provision to include a mixed delivery format and the consideration of time post‐stroke onset. This study also highlights the importance of considering the psychological impact of stroke prevention information in the acute stage.

### 4.1 Limitations and future directions

This study may be limited by the restriction of recruitment to a single hospital site and a single point in the recovery continuum. It is possible that needs may vary along the recovery continuum and between health sites. Further longitudinal research with multiple sites is required. As the focus of the study was on the experience of stroke education, two perspectives were included (i.e. stroke survivor and carer). It is possible that different information may have been obtained if additional perspectives, such as health professional interviews or chart audits of education provided, had been included (as per Salmon & Young, 2018). As time post‐stroke varied from 1 month to 4 years, there is the potential for recall bias. However, all participants reported remembering receiving stroke education. The study is also limited by the use of a single methodology, and the idea that some participants knew the researchers before the study while others did not which could have introduced a participation bias. A final limitation was that the study did not explore whether the education provided was culturally appropriate or the ethnicity or desired language of participants.

### 4.2 Clinical implications

The results of the current study provide valuable information for health professionals that provide stroke education as part of their role. The results highlight that there is no one‐size‐fits‐all approach to stroke education and that current stroke education does not fully meet the needs of stroke survivors and carers. Additionally, careful wording and phrasing of information when delivering stroke education were found to be essential, as some information about stroke prevention was perceived negatively while stroke survivors were still coming to terms with having a stroke. Health professionals could consider the potential need for redistribution of secondary stroke prevention information from the early stages post‐stroke, to a later stage in the recovery journey when stroke survivors may be more receptive to receiving information about secondary stroke prevention. It is essential that information about secondary stroke prevention is presented in a sensitive manner to ensure the continuity of hope for stroke survivors. The current study highlighted that stroke survivors and their carers should be included in the design, implementation and evaluation of stroke education programmes, beyond merely researching them. This may ensure that future stroke education is delivered in a format and timing that improves the emotional wellbeing of stroke survivors and carers rather than increasing their distress during this vulnerable time. Finally, while this study was conducted in Australia, the findings may be applicable to health services internationally.

## 5 CONCLUSION

Stroke survivors and carers expressed positive attitudes towards stroke education. However, participants highlighted a need for education to occur at multiple time points, in mixed formats and for content to be individualised. There is an urgent need to ensure that education for stroke survivors and their families is delivered with appropriate content, and in a way that is easily accessible, effective and meets their individual needs. Ensuring these elements are met may be an important step towards improving the rates and effectiveness of stroke education. The active inclusion of stroke survivors and their carers in the design, implementation and evaluation of stroke education programmes may be an important dimension for meeting the accessibility and acceptability issues raised in the current study. Further research is required to develop and trial accessible stroke education programmes based on the identified areas of need raised in the current study.

## AUTHOR CONTRIBUTIONS

Conceptualization: Emma Finch, Ashley Cameron, Katherine Jaques, Jennifer Lethlean, Darshan Shah, Christian Moro. Data collection: Emma Finch, Ellie Minchell. Data analysis and interpretation: Emma Finch, Ellie Minchell, Ashley Cameron, Katherine Jaques, Jennifer Lethlean, Darshan Shah, Christian Moro. Manuscript writing and editing: Emma Finch, Ellie Minchell, Ashley Cameron, Katherine Jaques, Jennifer Lethlean, Darshan Shah, Christian Moro.

## CONFLICT OF INTEREST

The authors report no conflicts of interest.

## Data Availability

The data that support the findings of this study are available on request from the corresponding author. The data are not publicly available due to privacy or ethical restrictions.
